# TorchGWAS2: Cost-Effective Phenome- and Genome-Wide Association Testing in Related Samples

**DOI:** 10.64898/2026.09.10.26362744

**Published:** 2026-09-16

**Authors:** Mengyu Zhang, Ziqian Xie, Samaneh Salehi Nasab, Nannan Wang, Xingzhong Zhao, Taryn Alkis, John Barnard, Thomas W. Blackwell, Russell P. Bowler, Shinhye Chung, Michael H. Cho, Clary B. Clish, Emily Drzymalla, Anne M. Evans, Nora Franceschini, Robert E. Gerszten, Madeline G. Gillman, Megan L. Grove, Nancy L. Heard-Costa, Scott R. Hutton, Rachel S. Kelly, Charles Kooperberg, Martin G. Larson, Jessica Lasky-Su, Deborah A. Meyers, Franklin P. Ockerman, Laura M. Raffield, Alexander P. Reiner, Stephen S. Rich, Jerome I. Rotter, Albert V. Smith, Kent D. Taylor, Ramachandran S. Vasan, Scott T. Weiss, Kari E. Wong, Alexis C. Wood, Prescott G. Woodruff, Lang Wu, Ronit I. Yarden, Junsun Yu, Laura Y. Zhou, Bing Yu, Degui Zhi, Han Chen

## Abstract

Modern large-scale genetic association analyses of imaging and omics data reveal unprecedented details of the genetic architecture of complex traits. Such analyses involve scanning thousands of phenotypes using linear mixed model-based genome-wide association study tools to control for sample relatedness. However, current LMM tools are not designed for such scale, creating a computational burden that hinders discovery. We propose TorchGWAS2, a cost-effective solution that overcomes the bottleneck using a deterministic variance-correction algorithm for LMMs, making it well-suited for GPU acceleration. TorchGWAS2 is applicable to unrelated and related individuals, cross-sectional and longitudinal studies, with and without missing data, and its computational complexity scales linearly with the number of phenotypes, genetic variants, and individuals. TorchGWAS2 showed more powerful association testing across 128 retinal image-derived endophenotypes of pairs of eyes from 64,703 UK Biobank participants and achieved two orders of magnitude speed-up analyzing 1,023 circulating metabolites in 16,352 Trans-Omics for Precision Medicine participants.

## Full Text Availability

The license terms selected by the author(s) for this preprint version do not permit archiving in PMC. The full text is available from the preprint server.

